# Efficacy of Fluoride Toothpaste Fortified With Micro‐ and Nano‐Hydroxyapatite on Remineralization and Surface Roughness of Synthetic Enamel

**DOI:** 10.1002/cre2.70471

**Published:** 2026-10-04

**Authors:** Porawit Kamnoedboon, Phakvalunch Rujiraprasert, Thawanrat Singthong, Thanaphum Osathanon, Chalermkwan Makornpan, Chawalid Pianmee, Naruporn Monmaturapoj, Dusit Nantanapiboon

**Affiliations:** ^1^ Faculty of Dentistry Chulalongkorn University Bangkok Thailand; ^2^ Clinic of Gerodontology and Special Care Dentistry, Center for Dental Medicine University of Zurich Zurich Switzerland; ^3^ Department of Operative Dentistry, Faculty of Dentistry Chulalongkorn University Bangkok Thailand; ^4^ Department of Cariology and Operative Dentistry, Graduate School of Medical and Dental Sciences Institute of Science Tokyo Tokyo Japan; ^5^ Dental Materials Research and Development Center, Faculty of Dentistry Chulalongkorn University Bangkok Thailand; ^6^ Oral Biology Research Center, Faculty of Dentistry Chulalongkorn University Bangkok Thailand; ^7^ Center of Excellence for Dental Stem Cell Biology and Department of Anatomy, Faculty of Dentistry Chulalongkorn University Bangkok Thailand; ^8^ National Metal and Materials Technology Center (MTEC) National Science and Technology Development Agency Pathum Thani Thailand; ^9^ Dental Center Surin Hospital Surin Thailand; ^10^ Center of Excellence and Innovation for Oral Health and Healthy Longevity and Center of Excellence for Dental Stem Cell Biology, Faculty of Dentistry Chulalongkorn University Bangkok Thailand

**Keywords:** calcium phosphate, geriatric, hydroxyapatite, microhardness, nano‐hydroxyapatite, remineralization, surface roughness, toothpaste

## Abstract

**Objectives:**

To evaluate the remineralization efficacy and surface effects of 1450 ppm fluoride toothpaste fortified with synthetic hydroxyapatite (Syn‐HAP) of varying particle sizes and concentrations on synthetic enamel.

**Material and Methods:**

Nano‐sized Syn‐HAP (nanoSyn‐HAP) was synthesized via a mechanochemical process and characterized using X‐ray diffraction (XRD) and transmission electron microscopy (TEM). Sixty disc‐shaped synthetic pellets underwent artificial caries induction and were randomly divided into five groups: Untreated (negative control), commercial 1450 ppm fluoride toothpaste (FT), FT plus 5% micrometer‐sized Syn‐HAP (FT+5mHAP), FT plus 5% nanoSyn‐HAP (FT+5nHAP), and FT plus 10% nanoSyn‐HAP (FT+10nHAP). Specimens then underwent a 15‐day pH cycling regimen with twice‐daily treatments. Knoop microhardness was evaluated at baseline, post‐caries induction, and post‐treatment; surface roughness and scanning electron microscopy (SEM) were assessed at baseline and post‐treatment. Data were analyzed using ANOVA and Bonferroni post‐hoc tests (*p* < 0.05).

**Results:**

XRD confirmed a pure hydroxyapatite phase, and TEM indicated that 93% of particles were smaller than 50 nm. All toothpaste‐treated groups exhibited significant microhardness recovery versus the untreated group (*p* < 0.05), with no significant differences among them. The untreated and FT groups showed significantly increased roughness after 15 days (*p* < 0.01), whereas all Syn‐HAP groups maintained smoothness comparable to baseline, with significantly lower roughness than FT (*p* < 0.01). SEM corroborated these findings, revealing smoother surfaces with particle integration in the Syn‐HAP groups.

**Conclusions:**

Under the conditions of this in vitro study, supplementing 1450 ppm fluoride toothpaste with Syn‐HAP produced surface microhardness recovery that did not differ significantly from that of standard fluoride toothpaste, while significantly improving the preservation of surface smoothness. Syn‐HAP‐fortified formulations therefore offer a promising approach for caries management, combining effective surface hardening with superior surface integrity, although confirmation in in situ and in vivo settings is required.

## 1 Introduction

Dental caries is a prevalent chronic disease affecting individuals of all ages. A systematic review reported that the global prevalence of dental caries is 46.2% in primary teeth and 53.8% in permanent teeth (Kazeminia et al. 2020). Caries etiology is multifactorial, driven by interactions between oral bacteria, fermentable carbohydrates, and tooth structure. The disease process initiates when cariogenic bacteria metabolize sugars to produce acids, leading to demineralization of the tooth surface. Without adequate remineralization, these lesions can progress into cavitation (Pitts et al. 2017). Regular brushing with fluoride toothpaste is the gold standard for caries prevention. Fluoride facilitates remineralization by binding with calcium and phosphate ions to form fluorapatite, which is more resistant to acid dissolution than natural hydroxyapatite (Cate 1999). However, fluoride intake requires careful monitoring, particularly in children under 6 years of age during amelogenesis. Excessive systemic fluoride exposure from toothpaste ingestion, estimated to range from 20% to 50% in young children (Petrović et al. 2023), can lead to dental fluorosis (Walsh et al. 2019; Wright et al. 2014).

To address safety concerns while maintaining enamel integrity, alternative remineralizing agents have been developed. Synthetic hydroxyapatite (Syn‐HAP), Ca_10_(PO_4_)_6_(OH)_2_, is a calcium phosphate biomaterial widely used in dentistry due to its biocompatibility and chemical resemblance to human tooth structure (Mondal et al. 2023). Syn‐HAP acts as a remineralizing agent by increasing the availability of calcium and phosphate ions on the tooth surface (Gjorgievska et al. 2013). Recent clinical trials indicate that toothpaste containing hydroxyapatite is non‐inferior to fluoride toothpaste in reducing cariogenic bacterial load (Campus et al. 2024) and preventing caries (Pawinska et al. 2024).

The particle size of Syn‐HAP may play a critical role in its efficacy. Previous studies suggest that nanometer‐sized Syn‐HAP (nanoSyn‐HAP) exhibits superior adhesion to the tooth surface compared to micrometer‐sized particles (microSyn‐HAP), likely due to differences in surface energy (Pushpalatha et al. 2023). While various synthesis techniques exist, such as co‐precipitation and sol‐gel methods (DileepKumar et al. 2022), the optimal particle size and concentration for dental applications remain to be definitively established. Furthermore, there is limited data on the combined remineralization efficacy of fluoride toothpaste supplemented with nano‐hydroxyapatite.

The present in‐vitro study aimed to assess the remineralization efficacy and surface effects of fluoride toothpaste fortified with Syn‐HAP of varying particle sizes. Specifically, this study compared an in‐house 1450 ppm fluoride toothpaste containing 5% or 10% nanoSyn‐HAP against a standard commercial 1450 ppm fluoride toothpaste and a formulation supplemented with microSyn‐HAP. The study utilized synthetic hydroxyapatite pellets as an enamel model to evaluate Knoop microhardness, surface roughness, and surface morphology following artificial demineralization. Synthetic hydroxyapatite pellets were chosen deliberately as the enamel model because they offer a chemically defined and compositionally uniform surface, eliminating the substantial variability associated with natural enamel, such as differences in donor age, tooth type, mineral density, and prior fluoride exposure. Because the composition of dental apatite varies between individuals and influences enamel behavior, standardized synthetic surfaces provide well‐defined and reproducible specimens that are advantageous for comparative laboratory research (Dudek et al. 2025). This uniformity provides a controlled baseline that improves the sensitivity of between‐group comparisons in a pH‐cycling design, and the material has been characterized and validated as an enamel substitute in dental materials research (Rujiraprasert et al. 2023). The null hypotheses tested were: (i) there would be no significant difference in microhardness recovery among the different toothpaste formulations, and (ii) there would be no significant difference in surface roughness among the treated groups.

## 2 Materials and Methods

### 2.1 Synthesis of NanoSyn‐HAP

NanoSyn‐HAP was synthesized using analytical reagent grade CaCO_3_ (Fluka, Darmstadt, Germany) and CaHPO_4_ (Sigma Aldrich, München, Germany). Deionized water (DI water) served as the medium for the synthesis process. Hydroxyapatite was synthesized via a mechanochemical process, wherein CaCO_3_ and CaHPO_4_ were mixed at a Ca/P molar ratio of 1.67. The raw materials were mixed by ball milling with zirconia balls for 40 h. The resulting slurry was dried at 100°C for 24 h to obtain hydroxyapatite powder.

### 2.2 Characterization of NanoSyn‐HAP

The phase composition of the as‐synthesized powder was analyzed using X‐ray diffraction (XRD; PANalytical X'Pert Pro, PANalytical, Almelo, The Netherlands) with CuK*α* radiation (*λ* = 1.5406 Å), operated at 30 mA and 40 kV. Scanning was performed from 20° to 60° 2*θ*, with a step size of 0.02° and a scanning speed of 2.4°/min. Spectra were analyzed using JADE software and compared with JCPDS card No. 09‐0432 to confirm the presence of hydroxyapatite.

Particle morphology and size were examined using transmission electron microscopy (TEM; JEM‐2010, JEOL, Tokyo, Japan). The powder was dispersed in ethanol, sonicated for 5 min, deposited onto a carbon‐coated grid, and dried. Imaging was conducted at 200 kV at magnifications ranging from 20,000× to 100,000×. Particle size and size distribution were determined using ImageJ software (version 1.53, National Institutes of Health, USA) after calibration against the scale bar of each micrograph. Only clearly defined particles with distinct boundaries were measured, and the diameter of each particle was recorded; particles within dense agglomerates whose boundaries could not be resolved were excluded. A total of 573 primary particles were measured across 22 micrographs. The particles were classified into three size ranges (1 to 50 nm, 51 to 100 nm, and greater than 100 nm), and the proportion in each range was calculated as a percentage of the total number measured, with the mean and standard deviation in each range reported in Table 2. The overall particle size distribution is shown in Figure 1d.

**Figure 1:**
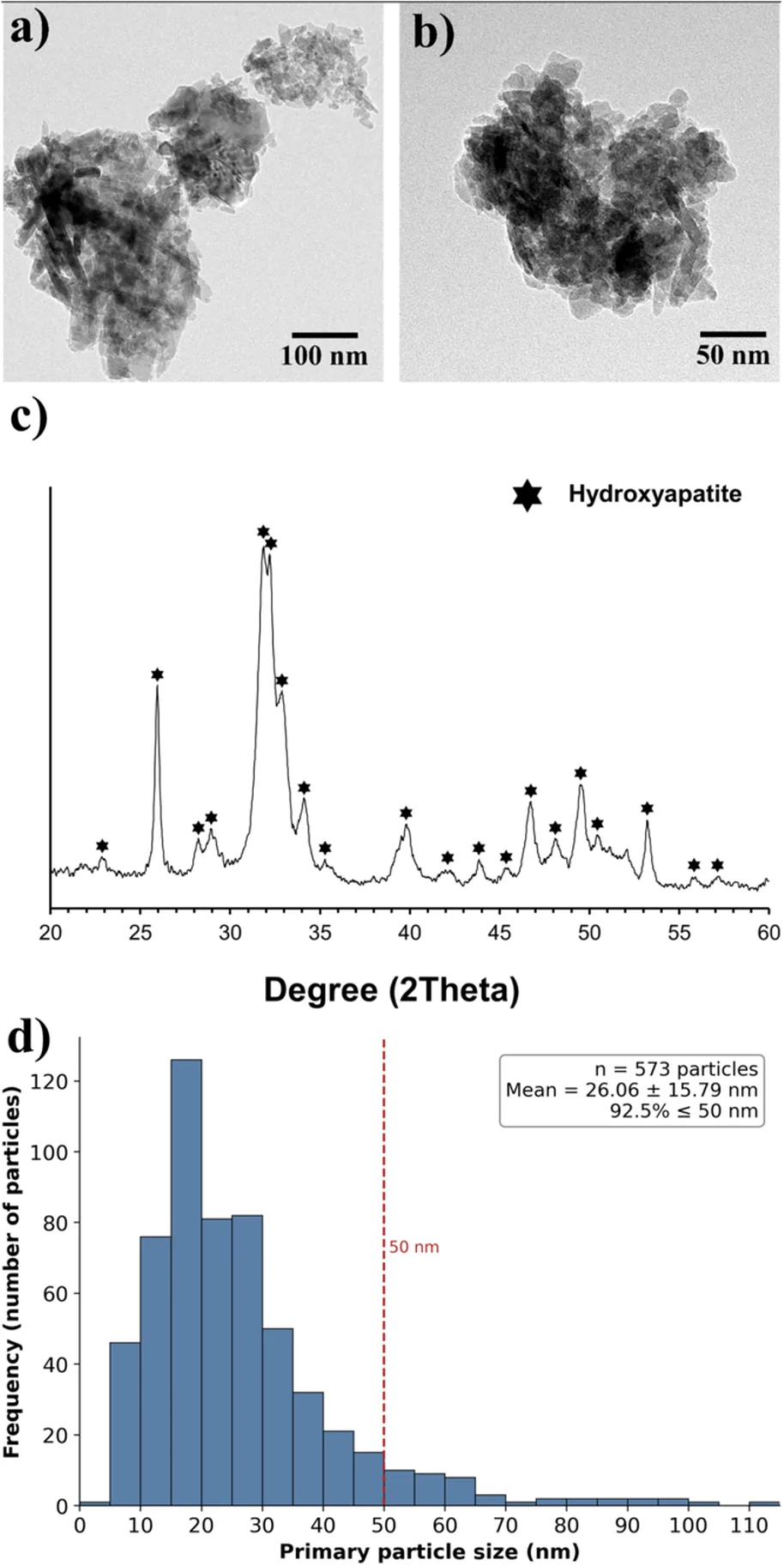
Characterization of the as‐synthesized nano‐hydroxyapatite. (a, b) TEM micrographs at magnifications of (a) 50,000× and (b) 100,000×. (c) XRD pattern of the as‐synthesized hydroxyapatite; the y‐axis is labeled Intensity (a.u.). (d) Particle size distribution of the synthesized nano‐hydroxyapatite (*n* = 573 primary particles measured from 22 TEM micrographs).

### 2.3 Sample Size Calculation

Ethical approval and informed consent were not required for this study, as it was limited to dental materials research and did not involve human tissues or patient participation. The sample size was calculated using G*Power software (version 3.1) based on reference values from a previous study by Juntavee et al. (2021). Parameters included a 95% confidence interval, 95% power, and an effect size of 0.96. The calculation indicated a minimum of three specimens per group; however, this study included 10 specimens per group to account for potential error.

### 2.4 Specimen Preparation

Synthetic hydroxyapatite pellets (NSTDA, Bangkok, Thailand) were used as an enamel substitute. This material was selected to ensure compositional consistency and a uniform, defect‐free surface geometry suitable for standardized microhardness and surface roughness measurement, while minimizing the biological variation inherent to natural enamel discs (e.g., variation in donor age, tooth type, mineral content, and previous fluoride exposure). Calcium phosphate ceramic of this type has been previously characterized and validated as a model enamel substitute material for dental applications (Rujiraprasert et al. 2023), and synthetic hydroxyapatite has been independently reported as a suitable, well‐defined substitute for natural enamel in standardized in vitro studies (Dudek et al. 2025). The pellets were produced from mechanochemically synthesized hydroxyapatite powder consolidated by uniaxial pressing and sintered at 1250°C, a material previously characterized in detail by our group (Rujiraprasert et al. 2023). In that characterization, X‐ray diffraction of the sintered material confirmed a single hydroxyapatite phase (JCPDS No. 09‐0432) with no minor phases, and scanning electron microscopy revealed a dense, homogeneous microstructure composed of well‐equiaxed hexagonal grains of approximately 5 µm. This equiaxed, isotropic polycrystalline structure differs fundamentally from the highly organized, rod‐based (prismatic) arrangement of crystals in natural enamel, and the synthetic substrate therefore provides a uniform surface without the rod and interrod architecture or the surface‐to‐core property gradient of human enamel. Sixty disc‐shaped pellets (6 mm diameter, 3 mm height) were embedded in epoxy resin (Rungart, Bangkok, Thailand). The exposed surfaces were wet‐polished using an automated machine (MINITECH 233, PRESI, France) with silicon carbide papers (grit 600‐2400, TOA, Thailand), followed by polishing with 0.05‐µm aluminum oxide particles to achieve a glossy finish. Specimens were cleaned in an ultrasonic cleaner (Bransonic, Germany) for 5 min and inspected under a 10× stereomicroscope (SZ 61, Olympus, Japan) to ensure they were defect‐free. Specimens were stored in an incubator (LK Lab, Korea) at 37°C and 100% relative humidity (Supporting Information S1).

### 2.5 Artificial Caries Formation

All prepared specimens were then immersed in a 0.1% lactic acid solution (pH 5.0) for 12 h at 37°C in the incubator to simulate artificial caries formation before the study group allocation.

### 2.6 Study Groups

Sixty specimens were randomly allocated into five groups (*n* = 12 per group): (i) Untreated: Negative control (Deionized water), (ii) FT: Commercial 1450 ppm fluoride toothpaste, (iii) FT+5mHAP: Commercial 1450 ppm fluoride toothpaste with 5% wt micrometer‐sized Syn‐HAP, (iv) FT+5nHAP: In‐house 1450 ppm fluoride toothpaste with 5% wt nanometer‐sized Syn‐HAP, (v) FT+10nHAP: In‐house 1450 ppm fluoride toothpaste with 10% wt nanometer‐sized Syn‐HAP. Ten specimens per group were designated for microhardness and surface roughness testing, while two were reserved for scanning electron microscopy (SEM). Detailed compositions are provided in Table 1.

**Table 1:** The code groups, composition of the tested toothpaste, batch numbers, and expiration dates in detail.

Products	Batch number and expiration date	Detailed compositions
FT (Fluoride 1450 ppm)	MGF140223TH112E EXP 140226	Commercial fluoride toothpaste (Colgate): Dicalcium phosphate dihydrate, water, sorbitol, sodium lauryl sulfate, cellulose gum, flavor, sodium monofluorophosphate (1450 ppm), sodium saccharin, tetrasodium pyrophosphate, sodium fluoride
FT+5mHAP (Fluoride 1450 ppm + 5% wt Micro‐hydroxyapatite)	MGF1001280922 EXP 280325	Commercial micro‐hydroxyapatite combined with fluoride toothpaste: Aqua (water), hydrated silica, sorbitol, glycerin, hydroxyapatite, cellulose gum, sodium monofluorophosphate (1450 ppm), sodium lauroyl sarcosinate, aroma (flavor), calcium carbonate, sodium benzoate, rebaudioside a, xylitol, limonene, menthol, mentha piperita (peppermint) oil, CI 42090 (Blue 1)
FT+5nHAP (Fluoride 1450 ppm + 5% wt Nanohydroxyapatite)	EXP: 21022025	In‐house toothpaste: Erythritol, hydrated silica, menthol, 5% wt nanohydroxyapatite, potassium sorbate, sodium benzoate, sodium carboxymethyl cellulose, sodium monofluorophosphate (1450 ppm), sodium lauryl sarcosinate, 70% sorbitol, sucralose, titanium dioxide, xanthan gum, CI4540, CI16035, flavor, water
FT+10nHAP (Fluoride 1450 ppm + 10% wt Nanohydroxyapatite)	EXP: 21022025	In‐house toothpaste: Erythritol, hydrated silica, menthol, 10% wt nanohydroxyapatite, potassium sorbate, sodium benzoate, sodium carboxymethyl cellulose, sodium monofluorophosphate (1450 ppm), sodium lauryl sarcosinate, 70% sorbitol, sucralose, titanium dioxide, xanthan gum, CI4540, CI16035, flavor, water
Deionized water	Prepare for 3–7 days before use	0 ppm, Dental Materials Research and Development Center, Faculty of Dentistry, Chulalongkorn University, Thailand
Demineralization solution	Prepare for 3–7 days before use	0.05 M acetic acid, 2.2 mM Ca(NO3)2, and 2.2 mM KH2PO4 in KOH buffer at pH 4.4
Remineralization solution	Prepare for 3–7 days before use	20 mmol/L HEPES, 1.5 mM CaCl_2_, 0.9 mM KH_2_PO_4_, 130 mM KCl, and 1 mM sodium azide (NaN_3_), pH adjusted to 7.0 using KOH
Artificial saliva	MFD: 12012023	0.75 g KCl, 0.07 g MgCl2, 0.199 g CaCl2, 0.965 g K2HPO4, 6 g sodium carboxymethylcellulose BP, 2.4 g sodium benzoate, and deionized water to a total of 1 liter

The hydroxyapatite concentrations of 5% and 10% by weight were selected on the basis of previous remineralization studies. A concentration of 10% nano‐hydroxyapatite has been widely adopted as an optimal level for the remineralization of early carious lesions and is the concentration most frequently used when hydroxyapatite toothpastes are compared with fluoride toothpastes (Amaechi et al. 2019; Juntavee et al. 2021). The 5% concentration was included both to evaluate a lower, dose‐dependent response and to match the concentration of the commercial micrometer‐sized hydroxyapatite comparator (FT+5mHAP), allowing a direct comparison of micro‐ and nano‐sized particles at an equivalent weight concentration. All hydroxyapatite percentages reported in this study represent weight ratios (% w/w), defined as grams of synthetic hydroxyapatite per 100 g of toothpaste. The micrometer‐sized hydroxyapatite (Omyadent 100‐OG, Omya International AG, Switzerland) had a median particle size (d50) of 5 to 9 µm and a top cut (d98) of 9 to 20 µm, according to the manufacturer's specification (laser diffraction, Malvern Mastersizer 2000).

### 2.7 pH Cycling and Remineralization Protocol

A toothpaste slurry was freshly prepared for each treatment group by mixing the toothpaste with distilled water in a 1:3 weight ratio, following the protocol of the previous study (Juntavee et al. 2021). The pH cycling regimen simulated daily oral conditions. The cycle was repeated twice daily for 15 days as follows: (i) Treatment 1: Specimens were immersed in the assigned toothpaste slurry for 4 min using an incubator shaker (Multitron, Inforsag, Switzerland) and were not rinsed immediately. (ii) Demineralization: Specimens were immersed in the demineralization solution (Table 1) for 3 h. (iii) Rinse and Remineralization: Specimens were rinsed with deionized water for 5 min, then submerged in the remineralization solution (Table 1) for 2 h. (iv) Treatment 2: Specimens were immersed in the toothpaste slurry again for 4 min. (v) Overnight: Specimens were stored in artificial saliva for 14 h.

All solutions were replaced daily. The untreated group underwent the pH cycling regimen but was immersed in deionized water instead of toothpaste slurry.

### 2.8 Microhardness and Surface Roughness Tests

Knoop microhardness was measured using a microhardness tester (FM810, Future‐Tech Corp., Japan) with a 100‐g load applied for 15 s. Measurements were recorded at three specific time points: baseline (sound synthetic enamel surfaces; KHN0), after artificial caries formation (KHN1), and after the 15‐day pH cycling and treatment regimen (KHN15). Five indentations were made per specimen, positioned 200 µm from the border. Surface microhardness recovery (%SMHR) was calculated (Supporting Information S2).

Surface roughness (*R*_a_) was evaluated at baseline and after the 15‐day pH‐cycling and treatment period using a non‐contact optical profilometer (InfiniteFocus SL, Alicona, Graz, Austria) at 50× magnification, with a scan area of 0.4 by 0.4 mm per measurement. Five locations were measured on each specimen at each time point (five at baseline and five after 15 days), each positioned approximately 500 µm from the microhardness indentations. The arithmetic mean roughness (*R*_a_, a line parameter) was recorded in nanometers, and the mean of the five measurements was used to represent each specimen.

An additional two specimens from each group after 15 days, plus two sound baseline specimens, were analyzed using SEM (Quanta 250, FEI, USA). Samples were sputter‐coated with gold and observed at 5000× and 10,000× magnification (20 kV).

### 2.9 Statistical Analysis

Data were analyzed using IBM SPSS Statistics version 29.0.1.0. Differences in microhardness were analyzed using repeated measures ANOVA and one‐way ANOVA, each followed by Bonferroni post hoc tests. Surface roughness was compared using one‐way ANOVA with Bonferroni post hoc tests and paired *t*‐tests. Significance was set at *p* < 0.05.

## 3 Results

### 3.1 Characterization of NanoSyn‐HAP

The X‐ray diffraction (XRD) pattern of the as‐synthesized powder confirmed the phase composition as pure hydroxyapatite (Figure 1). The characteristic peaks were identified at 2*θ* of 31.9°, matching JCPDS standard No. 09‐0432. No impurity phases were detected. TEM revealed that the individual primary particles were predominantly nanometer‐sized but were largely present as agglomerates of these primary particles, an expected characteristic of nano‐sized hydroxyapatite attributable to its high specific surface area and surface energy (Joudeh and Linke 2022). Based on measurement of 573 primary particles, approximately 93% (535 of 573) were smaller than 50 nm, and this dominant sub‐50 nm fraction had a mean size of 23.01 ± 10.39 nm; the overall mean size of all measured particles was 26.06 ± 15.79 nm (Table 2 and Figure 1d). The reported distribution therefore refers to the dimensions of the primary particles rather than to the size of the agglomerates.

**Table 2:** Particle size distribution of nano‐hydroxyapatite.

	Particle size range		
	1–50 nm	51–100 nm	> 100 nm
Average	23.01 ± 10.39	66.91 ± 13.75	107.38 ± 7.90
%	93.37	6.28	0.35

*Note:* Values were obtained by measurement of individual primary particles from TEM micrographs; percentages represent the proportion of the total number of particles measured in each size range.

### 3.2 Microhardness Test

At baseline (Figure 2 and Table 3), no significant differences in microhardness were observed among the groups (*p* = 1.00). Following artificial caries formation, microhardness values in all groups significantly decreased compared to baseline (*p* < 0.05). Additionally, there were no significant differences in KHN values among the groups at the post‐induced artificial caries stage (*p* = 1.00), indicating uniform lesion formation across all specimens.

**Figure 2:**
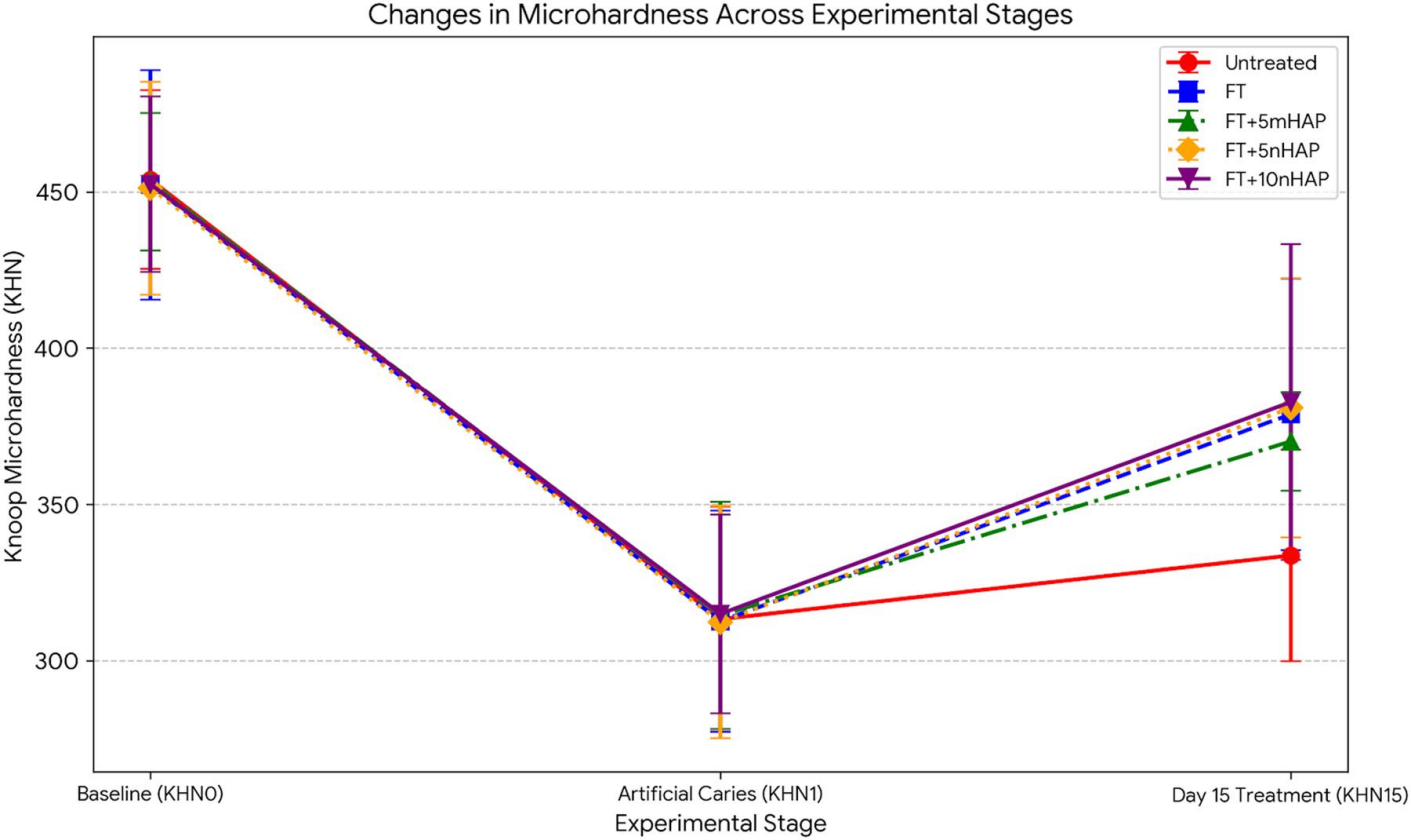
Changes in Knoop microhardness (KHN) across experimental stages. The line plot illustrates the mean Knoop microhardness values ± standard deviation (SD) for each experimental group (*n* = 10) at three time points: Baseline (sound synthetic enamel), artificial caries formation (post‐demineralization), and after 15 days of pH cycling and treatment. Groups: Untreated (negative control), FT (1450 ppm fluoride toothpaste), FT+5mHAP (1450 ppm fluoride + 5% micro‐hydroxyapatite), FT+5nHAP (1450 ppm fluoride + 5% nano‐hydroxyapatite), and FT+10nHAP (1450 ppm fluoride + 10% nano‐hydroxyapatite). Error bars represent the standard deviation.

**Table 3:** The Knoop microhardness (KHN) values (mean ± SD) and the percentage of surface microhardness recovery (%SMHR).

Groups	Baseline (KHN0)	Artificial caries formation (KHN1)	Day 15 after the pH cycling and the treatment (KHN15)	Average %SMHR ± SD
Untreated	453.92 ± 28.59^A,a^	313.27 ± 35.94^B,a^	333.66 ± 33.88^B,a^	4.52 ± 22.08
FT	452.24 ± 36.77^A,a^	312.67 ± 35.39^B,a^	378.81 ± 43.41^C,b^	41.82 ± 19.69
FT+5mHAP	453.25 ± 21.96^A,a^	314.49 ± 36.38^B,a^	370.14 ± 15.81^C,b^	37.39 ± 14.35
FT+5nHAP	451.19 ± 34.05^A,a^	312.32 ± 37.2^B,a^	380.89 ± 41.43^C,b^	45.11 ± 27.49
FT+10nHAP	452.47 ± 28.09^A,a^	314.96 ± 31.8^B,a^	382.73 ± 50.48^C,b^	58.74 ± 15.00

*Note:* Different superscript uppercase letters indicate statistically significant differences among different time points in the same row (repeated measures ANOVA, Bonferroni post hoc). Different superscript lowercase letters indicate statistically significant differences among study groups in the same column (one‐way ANOVA, Bonferroni post hoc).

After the 15‐day pH cycling regimen, all treatment groups (FT, FT+5mHAP, FT+5nHAP, and FT+10nHAP) exhibited a significant increase in microhardness compared to the post‐demineralization values (*p* < 0.05). In contrast, the untreated group showed no significant recovery (*p* = 1.00).

When comparing groups at Day 15, all toothpaste‐treated groups demonstrated significantly higher microhardness than the untreated group (*p* < 0.05). However, no statistically significant differences were found among the toothpaste formulations themselves (FT vs. FT+5mHAP vs. FT+5nHAP vs. FT+10nHAP). The highest %SMHR was observed in the FT+10nHAP group (58.74%), followed by FT+5nHAP (45.11%), FT (41.82%), and FT+5mHAP (37.39%).

### 3.3 Surface Roughness Test

At baseline (Table 4), all groups exhibited comparable surface roughness (*p* = 0.998). After 15 days, a significant increase in surface roughness was observed in the untreated group (*p* < 0.01) and the FT group (*p* < 0.01). Conversely, the surface roughness values for the FT+5mHAP, FT+5nHAP, and FT+10nHAP groups did not change significantly from baseline (*p* > 0.05).

**Table 4:** The surface roughness (*R*_a_) values (mean ± SD, in nanometers) at baseline and after 15 days of treatment.

Groups	Baseline before the artificial caries induction (nm)	Day 15 after the pH cycling and the treatment (nm)
Untreated	268.55 ± 23.33^A,a^	488.02 ± 17.23^B,a^
FT	266.29 ± 20.24^A,a^	339.16 ± 43.91^B,b^
FT+5mHAP	269.56 ± 32.59^A,a^	255.56 ± 34.63^A,c^
FT+5nHAP	265.97 ± 33.23^A,a^	259.25 ± 21.53^A,c^
FT+10nHAP	266.31 ± 24.18^A,a^	265.16 ± 28.12^A,c^

*Note:* Different superscript uppercase letters indicate statistically significant differences among different time points in the same row (repeated measures ANOVA, Bonferroni post hoc). Different superscript lowercase letters indicate statistically significant differences among study groups in the same column (one‐way ANOVA, Bonferroni post hoc).

Inter‐group comparison at Day 15 revealed that the untreated and FT groups had significantly higher surface roughness compared to the HAP‐containing groups (*p* < 0.01). The FT+5mHAP, FT+5nHAP, and FT+10nHAP groups showed no significant differences among each other (*p* > 0.05), indicating that the addition of HAP helped maintain surface smoothness relative to the control.

### 3.4 Surface Morphology Evaluation

Before pH cycling, all specimens displayed smooth surfaces with no signs of cavitation (Figure 3). After 15 days of the pH cycling regimen, the untreated group exhibited severe surface degradation, characterized by large lesions and visible cavitation. The FT group showed a smoother surface than the untreated group but still presented with small cavitations and some surface irregularities.

**Figure 3:**
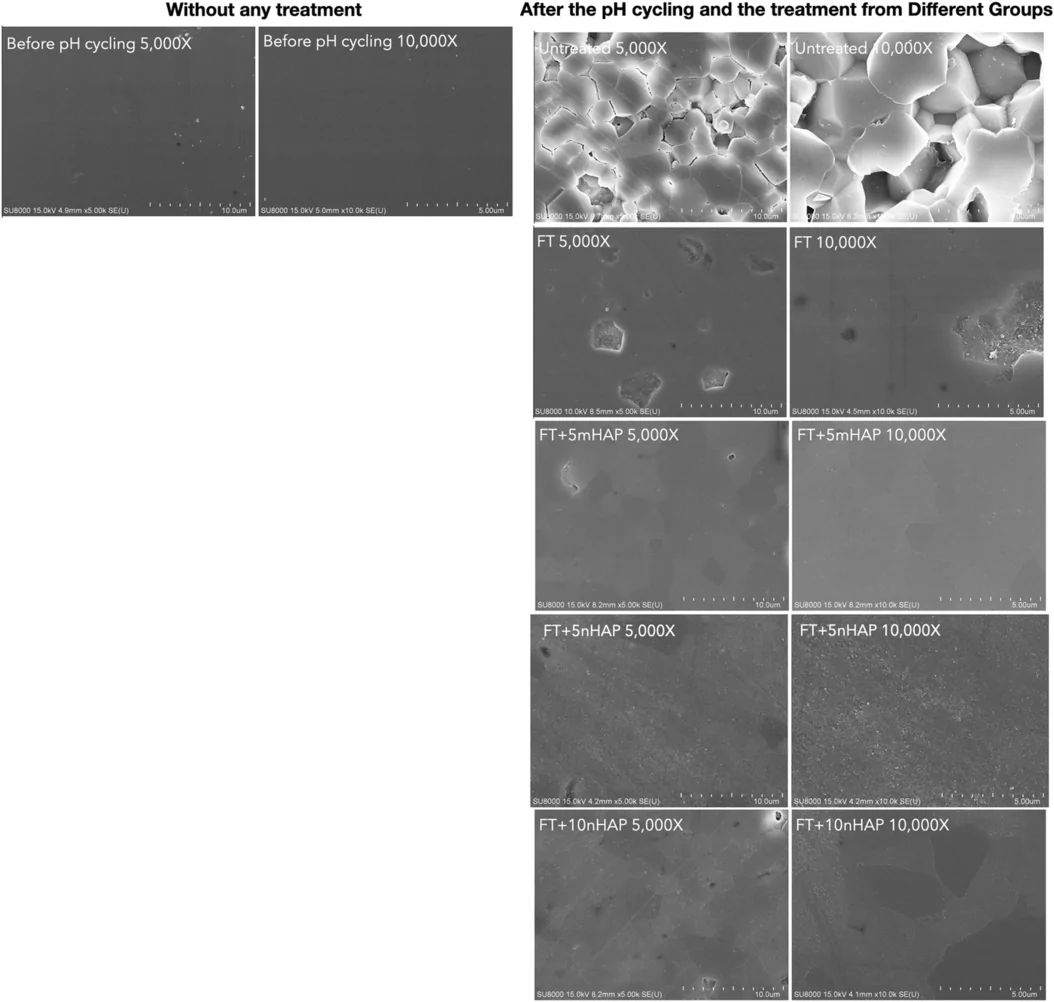
SEM photomicrographs of specimens without any treatment and after the pH cycling and treatment from different groups at 5000× and 10,000× magnifications.

In contrast, the groups treated with HAP‐containing toothpaste (FT+5mHAP, FT+5nHAP, and FT+10nHAP) exhibited smooth, non‐cavitated surfaces. In the nanoSyn‐HAP groups (5% and 10%), particles appeared to be embedded in the surface layer, contributing to a more homogeneous surface morphology compared to the FT and untreated groups.

## 4 Discussion

This study evaluated whether supplementing a 1450 ppm fluoride toothpaste with synthetic hydroxyapatite (Syn‐HAP) of different particle sizes and concentrations improves remineralization outcomes on a synthetic enamel model subjected to artificial caries formation and pH cycling. Two outcomes were emphasized: (i) Mineral recovery assessed by Knoop microhardness and (ii) surface quality assessed by roughness and SEM morphology. Overall, all treated groups demonstrated significantly greater microhardness after 15 days than the untreated control, confirming that the interventions promoted mineral recovery under the experimental conditions. However, microhardness did not differ significantly among fluoride alone and Syn‐HAP‐supplemented fluoride formulations, supporting acceptance of the first null hypothesis. In contrast, Syn‐HAP‐containing groups recovered surface roughness values comparable to baseline and exhibited significantly lower roughness than fluoride alone and untreated controls, leading to rejection of the second null hypothesis.

Fluoride toothpaste is a well‐established caries‐preventive intervention because fluoride adsorbs to enamel, forms a labile fluoride reservoir, and facilitates fluorapatite formation during remineralization challenges (Buzalaf et al. 2011). Consistent with this mechanism, fluoride‐treated specimens in the present study showed significant microhardness recovery compared with the untreated group after 15 days. Importantly, supplementation of fluoride toothpaste with either microSyn‐HAP or nanoSyn‐HAP did not produce a statistically significant additional increase in microhardness beyond fluoride alone. This pattern aligns with previous pH‐cycling studies in which fluoride and nano‐hydroxyapatite toothpastes both increased hardness relative to water controls but did not consistently differ from each other (Juntavee et al. 2021; Mielczarek and Michalik 2014). Moreover, as reported elsewhere, hardness values in treated groups did not return to baseline, suggesting incomplete remineralization of deeper or more porous regions of the lesion within the time frame of the model (Juntavee et al. 2021; Mielczarek and Michalik 2014). This “partial recovery” is expected because surface rehardening can occur earlier than full restoration of the internal lesion body.

Several explanations may account for the absence of microhardness differences among fluoride and fluoride+Syn‐HAP groups. First, a ceiling effect may occur when 1450 ppm fluoride already provides substantial remineralization under frequent cycling and slurry exposure; incremental benefits from additional calcium‐phosphate sources may be difficult to detect using surface microhardness alone. Second, Syn‐HAP in slurry form may preferentially contribute to surface deposition and smoothing rather than deeper mineral infiltration measurable by Knoop indentation, especially if particle agglomeration limits penetration into subsurface porosities. Third, the synthetic enamel substrate used in this study, while advantageous for standardization, may differ from natural enamel in microstructure and baseline mechanical properties; this could influence lesion development and the sensitivity of hardness testing to detect subtle between‐group differences.

From a mechanistic standpoint, nano‐sized calcium phosphate particles are often expected to enhance remineralization because smaller particles have greater surface area and higher reactivity, potentially increasing ionic availability and accelerating precipitation kinetics (Ginebra et al. 2004; Joudeh and Linke 2022). Additionally, fluoride and hydroxyapatite could theoretically act synergistically by combining fluoride‐driven crystal stabilization with a biomimetic mineral source. Despite these expectations, the current results suggest that, under the present protocol, Syn‐HAP supplementation did not measurably increase surface microhardness beyond fluoride alone. This is not inconsistent with the broader literature, where synergy appears more prominent in some delivery formats (e.g., rinses) than in others. For example, a fluoride mouth rinse combined with nano‐hydroxyapatite increased enamel microhardness more than fluoride alone and showed dose‐related effects (Kim et al. 2007). Differences in vehicle (rinse vs. toothpaste), exposure time, agitation, particle dispersion, and the presence/absence of abrasives may help explain why additive effects are observed in certain systems but not others.

Unlike microhardness, surface roughness clearly differentiated the formulations. Roughness increased significantly in the untreated group and also increased in the fluoride‐only group, whereas Syn‐HAP‐containing fluoride groups recovered roughness values close to baseline. This divergence suggests that Syn‐HAP contributes meaningfully to surface topography stabilization, even when microhardness recovery is similar.

The roughness increase in the untreated group is consistent with mineral dissolution and surface defect development during repeated acidic challenge. Organic acids can interact with apatite by binding calcium, promoting dissolution, and creating surface pits and irregularities (Yoshida et al. 2001), which corresponds to increased roughness after demineralization cycles and has been observed in other enamel demineralization models (Larnani et al. 2025). In the fluoride‐only group, increased roughness may reflect surface precipitation phenomena rather than continued dissolution alone. Fluoride can form calcium fluoride‐like deposits on enamel surfaces that function as reservoirs (Buzalaf et al. 2011). Such deposits may accumulate superficially and alter surface texture, potentially increasing measured roughness depending on deposit morphology and distribution. Prior work has reported that fluoride‐treated enamel surfaces can show altered topography and reduced nanomechanical properties compared with sound enamel, and that some surface changes may be reduced after cleaning/ultrasonication, consistent with removal of loosely bound superficial deposits (Jeng et al. 2008). The present SEM observations of surface particles in the fluoride group are compatible with this interpretation, although compositional confirmation would require elemental analysis.

In contrast, Syn‐HAP‐supplemented groups demonstrated smoother, more homogeneous surfaces on SEM and stable roughness values. Hydroxyapatite has been proposed to form a protective layer by adhering to enamel and creating mineral bridges that fill superficial defects, thereby improving surface regularity (Meyer et al. 2022). Moreover, nano‐hydroxyapatite can act as a template for apatite growth and promote crystal precipitation on demineralized surfaces (Amaechi and van Loveren 2013). Studies have similarly described a coating‐like layer after nano‐hydroxyapatite exposure that appears less crystalline than native enamel apatite (Roveri et al. 2009), which could be consistent with a biomimetic deposition process. In the present study, the persistence of low roughness in Syn‐HAP groups suggests that hydroxyapatite particles, whether micro‐ or nano‐sized, may reduce surface irregularities produced during cycling and/or modify the nature of surface deposits, resulting in a more uniform surface than fluoride alone.

Notably, no significant difference in surface roughness or microhardness recovery was detected between the nanometer‐sized and micrometer‐sized Syn‐HAP groups, despite a difference in particle size of approximately two to three orders of magnitude (a mean primary particle size of about 26 nm for the nanoSyn‐HAP vs. a median size of 5 to 9 µm for the micrometer‐sized hydroxyapatite). Several factors may account for this. First, as shown by TEM, the nanoSyn‐HAP primary particles were largely present as agglomerates; this agglomeration increases the effective functional size of the nanoparticles within the toothpaste slurry and may diminish the high surface area and reactivity normally expected of nanoscale material, narrowing the practical difference between the two formulations. Second, both micrometer‐ and nanometer‐sized hydroxyapatite supply calcium and phosphate ions and can deposit onto the surface to fill defects and form a coating layer; over the 15‐day period and at the matched 5% concentration used here, this surface‐level deposition appears to be governed more by the vehicle and the pH‐cycling conditions than by particle size. Third, the outcomes assessed here, namely surface roughness, surface microhardness, and surface morphology, primarily reflect near‐surface changes, and any size‐dependent differences in subsurface lesion remineralization may not be captured by these surface‐sensitive measurements; their detection would require cross‐sectional methods such as transverse microradiography or cross‐sectional microhardness. Together, these factors offer a plausible explanation for the comparable performance of the micrometer‐ and nanometer‐sized formulations under the present experimental conditions.

A practical contribution of this work is the use of mechanochemical synthesis (ball milling) to produce nanoSyn‐HAP suitable for incorporation into an in‐house fluoride toothpaste. Compared with precipitation, hydrothermal, or sol‐gel routes, mechanochemical processing can reduce synthesis complexity by avoiding pH adjustment and washing steps, which may improve scalability and yield (Dinda et al. 2020; Do and Friščić 2017). In the present study, the synthesized particles were predominantly < 50 nm, with a mean size around 23 nm, indicating successful nanoscale production. While the clinical performance of hydroxyapatite‐containing formulations depends on multiple factors beyond particle size alone (e.g., dispersion stability, abrasivity, and delivery mode), demonstrating a feasible synthesis approach supports future formulation optimization and translational manufacturing considerations.

This study benefits from a controlled pH‐cycling design, standardized specimen preparation, and complementary outcome measures (microhardness, roughness, SEM). However, several limitations should be considered when interpreting clinical implications. First, although synthetic hydroxyapatite pellets provide material standardization, reproducibility, and a uniform surface that strengthens the internal validity of between‐group comparisons (Dudek et al. 2025; Rujiraprasert et al. 2023), they do not fully replicate the properties of natural enamel. Natural enamel possesses a hierarchical prismatic architecture, a residual organic matrix, and a gradient of composition and microhardness from the outer surface toward the dentinoenamel junction, none of which are captured by a homogeneous ceramic pellet. As previously reported for this material (Rujiraprasert et al. 2023), the fabrication process does not reproduce the enamel rods aligned perpendicular to the dentinoenamel junction or the decussating Hunter‐Schreger bands, an arrangement that cannot currently be reproduced in the laboratory, and the sintered hydroxyapatite substrate possesses higher microhardness than natural enamel. In addition, natural enamel exhibits regional variation in mineral density and microhardness, both across the crown and through its thickness, and standardized indentation protocols are required to account for this heterogeneity (Okamoto et al. 2024). The synthetic pellet model used here intentionally eliminates this heterogeneity to improve between‐group comparability, but as a consequence it does not reproduce the depth‐dependent and site‐dependent mechanical behavior of natural enamel. Consequently, baseline hardness, lesion formation dynamics, and the pattern of mineral uptake may differ from those of human enamel, which limits the direct extrapolation of these in vitro findings to the clinical situation. Second, surface microhardness primarily reflects near‐surface changes and may not capture remineralization within the lesion body. Cross‐sectional methods (e.g., transverse microradiography, micro‐CT, or cross‐sectional microhardness) would strengthen conclusions regarding subsurface remineralization and could reveal differences not detected by surface Knoop testing. Third, the protocol relied on immersion in toothpaste slurry (ISO‐based preparation) rather than toothbrushing. Immersion may promote superficial precipitation and retention of loosely bound deposits, whereas brushing introduces shear forces and may enhance penetration into porous regions while also removing surface reservoirs. Future work should incorporate brushing simulation and abrasion/wear testing to clarify the persistence and functional significance of the observed surface layers. Fourth, the proposed explanations for fluoride‐related deposits versus hydroxyapatite‐mediated smoothing are plausible but indirect. Elemental analysis (EDS), phase analysis, and nanomechanical testing of the surface layer (e.g., nanoindentation) would help distinguish calcium fluoride‐like deposits from apatite‐rich coatings and quantify their mechanical integrity.

A further limitation concerns the characterization of the hydroxyapatite within the final formulations. X‐ray diffraction was performed on the synthesized nanoSyn‐HAP powder but not after its incorporation into the toothpaste, and formulation stability over time was not evaluated; we therefore cannot directly confirm that the hydroxyapatite crystal structure was fully retained after formulation or during storage. On mechanistic grounds, however, substantial conversion within the formulation is not expected, because the fluoride in both the commercial and the in‐house formulations is supplied predominantly as sodium monofluorophosphate, in which fluoride is covalently bound and is therefore not freely available to react with the calcium and phosphate of hydroxyapatite during storage. This is consistent with the established compatibility of monofluorophosphate with calcium‐based dentifrice systems and with the existence of commercial combined hydroxyapatite and monofluorophosphate toothpastes, including the micrometer‐sized comparator used here. Fluorapatite formation is expected to occur predominantly at the tooth surface during use, once monofluorophosphate is hydrolyzed, rather than within the formulation. Post‐incorporation phase analysis (X‐ray diffraction or Fourier‐transform infrared spectroscopy) and time‐course stability testing would be needed to confirm this directly, and are recommended for future work.

An additional limitation relates to the demineralization model. Artificial caries was induced chemically using lactic acid, and the pH‐cycling challenge used an acetic acid‐based demineralization solution. In contrast, caries‐associated demineralization in vivo results from a complex mixture of organic acids, including lactic, acetic, propionic, formic, and butyric acids, produced by a cariogenic biofilm in a dynamic environment that also involves the salivary pellicle, bacterial buffering, and fluctuating mineral saturation (Pitts et al. 2017). The buffering capacity and ionic composition contributed by this acid mixture influence the rate and pattern of mineral dissolution in ways that a single‐acid or single‐solution chemical model does not fully reproduce. Although the present model is standardized and widely used, it may not capture the full demineralization dynamics of natural caries, and future studies employing microbial or biofilm‐based caries models, or multi‐acid demineralization systems, would provide a more clinically representative challenge.

From a clinical perspective, maintaining a smoother surface after repeated demineralization and remineralization cycles is relevant because surface roughness is closely associated with bacterial adhesion and plaque retention (McConnell et al. 2010; Quirynen and Bollen 1995). A threshold roughness of approximately *R*_a_ 0.2 µm has been proposed, below which no further reduction in bacterial accumulation is expected, whereas increases above this threshold are accompanied by greater plaque accumulation and a correspondingly higher risk of caries and periodontal inflammation (Bollenl et al. 1997). In the present study, the untreated and fluoride‐only groups showed a marked increase in surface roughness after pH cycling (to approximately 0.49 µm and 0.34 µm, respectively), moving further above this threshold, whereas the Syn‐HAP‐containing groups remained close to their baseline values (approximately 0.26 µm). Although the absolute roughness of all groups, including the Syn‐HAP groups, remained somewhat above the proposed 0.2 µm threshold, which likely reflects the grain and pore structure of the sintered ceramic substrate, the ability of Syn‐HAP to prevent the additional acid‐induced roughening is clinically meaningful: by limiting the progressive increase in roughness, Syn‐HAP supplementation may help reduce plaque accumulation and the associated risk of secondary caries relative to fluoride alone. Therefore, even in the absence of additional microhardness gains over fluoride alone, Syn‐HAP supplementation may offer practical surface‐level benefits that warrant further investigation in more clinically representative settings, including in situ and in vivo models, longer‐term acid challenges, and assessments of deposit stability and acid resistance.

## 5 Conclusion

Within the limitations of this in vitro study, the following conclusions can be drawn. First, all toothpaste‐treated groups, including the standard 1450 ppm fluoride toothpaste and the formulations supplemented with micrometer‐ and nanometer‐sized synthetic hydroxyapatite (Syn‐HAP), produced significantly greater recovery of surface microhardness after artificial caries formation than the untreated control. No statistically significant difference in microhardness recovery was detected among the fluoride and Syn‐HAP groups. Because the study was not designed as an equivalence trial, this result indicates that Syn‐HAP supplementation did not impair the microhardness recovery achieved with fluoride, rather than establishing formal equivalence between the formulations. Second, the Syn‐HAP‐containing formulations were significantly more effective than standard fluoride toothpaste alone in preserving surface smoothness: whereas the fluoride‐only group showed increased surface roughness, likely associated with calcium fluoride‐like surface deposition, the Syn‐HAP groups maintained roughness values close to baseline.

Taken together, these findings suggest that fluoride toothpaste fortified with Syn‐HAP is a promising approach for caries management, providing surface microhardness recovery that did not differ significantly from that of standard fluoride toothpaste under the conditions tested, together with superior preservation of surface smoothness. These in vitro findings should be confirmed in clinically representative in situ and in vivo studies.

## Author Contributions

**Porawit Kamnoedboon:** conceptualization, methodology, investigation, data curation, writing – original draft, writing – review and editing. **Phakvalunch Rujiraprasert:** methodology, formal analysis, investigation, data curation. **Thawanrat Singthong:** methodology, formal analysis, investigation. **Thanaphum Osathanon:** writing – original draft, writing – review and editing. **Chalermkwan Makornpan:** methodology. **Chawalid Pianmee:** conceptualization, writing – original draft, writing – review and editing. **Naruporn Monmaturapoj:** writing – original draft, writing – review and editing. **Dusit Nantanapiboon:** conceptualization, methodology, resources, writing – review and editing, supervision, funding.

## Ethics Statement

This experiment did not require ethical approval because it did not involve human tissues or clinical procedures.

## Conflicts of Interest

The authors declare no conflicts of interest.

## Supporting information


Supporting File

## Data Availability

The data that support the findings of this study are available from the corresponding author upon reasonable request.
