# Supplementary material for: Efficacy of Fluoride Toothpaste Fortified With Micro‐ and Nano‐Hydroxyapatite on Remineralization and Surface Roughness of Synthetic Enamel
Source: Clin Exp Dent Res. 2026 Oct 4;12(5):e70471. doi: 10.1002/cre2.70471 (PMC13634605; doi:10.1002/cre2.70471)
Supplement: Supplementary file 1 [file CRE2-12-e70471-s001.docx]

**Supplementary Material 1**

**Supplementary Material 2**
